# Body fatness and physical activity at young ages and the risk of breast cancer in premenopausal women

**DOI:** 10.1038/sj.bjc.6602758

**Published:** 2005-09-13

**Authors:** C M K Magnusson, A W Roddam, M C Pike, C Chilvers, B Crossley, C Hermon, K McPherson, J Peto, M Vessey, V Beral

**Affiliations:** 1 Cancer Research UK Epidemiology Unit, University of Oxford, Richard Doll Building, Roosevelt Drive, Headington, Oxford OX3 7LF, UK; 2 Department of Public Health Sciences, Division of Social Medicine, Karolinska Institute, 171 76 Stockholm, Sweden; 3 Norris Comprehensive Cancer Center, Keck School of Medicine, Department of Preventive Medicine, University of Southern California, Los Angeles, CA 90033, USA; 4 Department of Health, E Floor, Government Office for the East Midlands, The Belgrave Centre, Stanley Place, Talbot Street, Nottingham NG1 5GG, UK; 5 Nuffield Department of Obstetrics and Gynaecology, University of Oxford, Womens Centre, Level 3, John Radcliffe Hospital, Headington, Oxford OX3 9DU, UK; 6 Cancer Research UK Section of Epidemiology, Brookes Lawley Building, Institute of Cancer Research, Sutton, Surrey SM2 5NG, UK; 7 Department of Epidemiology and Population Health, London School of Hygiene and Tropical Medicine, Keppel Street, London WC1E 7HT UK; 8 Unit of Health Care Epidemiology, Department of Public Health, Old Road Campus, Headington, Oxford OX3 7LF, UK

**Keywords:** childhood, adolescence, body fatness, participation in sports, premenopausal, breast cancer

## Abstract

We examined the relationship between body fatness, sports participation and breast cancer risk in 1560 premenopausal cases and 1548 controls, from three related population-based case–control studies in the UK. Half of the women with breast cancer were aged less than 36 years at diagnosis. Women who perceived themselves as plump at age 10 years had a relative risk of 0.83 (95% confidence interval 0.69–0.99, *P*=0.03) as compared with those who perceived themselves as thin. Self-reported obesity compared with leanness at diagnosis was associated with a relative risk of 0.78 (95% confidence interval 0.56–1.06, *P*=0.11). Women who reported having been plump at age 10 years and overweight or obese at diagnosis had a relative risk of 0.75 (95% confidence interval 0.56–1.01, *P*=0.06) as compared with those who reported being thin at age 10 years and at diagnosis. Findings for three related measures of body fatness suggested that obesity is associated with a reduced risk of premenopausal breast cancer. There was no association between sports participation and breast cancer risk in these premenopausal women. The relative risk for spending an average of more than 1 h per week in sports compared with less from ages 12 to 30 years was 1.00 (95% CI 0.86–1.16, *P*=0.98).

Obesity in adulthood has been related to a decreased risk of premenopausal breast cancer, but data on the relationship with body fatness in childhood and adolescence are limited (IARC, 2002; Okasha *et al*, 2003). Although an expert group convened by the International Agency for Research on Cancer concluded that there was sufficient evidence that physical activity reduced the risk of premenopausal breast cancer (IARC, 2002), results from more recent studies have been contradictory (Adams-Campbell *et al*, 2001; Gilliland *et al*, 2001; Matthews *et al*, 2001; Colditz *et al*, 2003; Dorn *et al*, 2003; Hirose *et al*, 2003; John *et al*, 2003; Steindorf *et al*, 2003; Yang *et al*, 2003; Margolis *et al*, 2005).

We report here on the relationship between premenopausal breast cancer, body fatness at age 10 years and in adulthood, and sports participation during puberty, late adolescence and early adulthood from three related UK case–control studies.

## MATERIAL AND METHODS

### Study population

This investigation includes data from three related UK population-based case–control studies of breast cancer of a similar design: cases were diagnosed between 1 January 1982 and 31 December 1985, below age 36 years in the first (UK National Case-Control Study Group, 1989); between 1 January 1988 and 30 June 1989 at ages 36–45 years in the second; and between 1 July 1990 and 30 June 1991 at ages 46–54 years in the third (unpublished data). Residents of many parts of England but not East Anglia in Greater Glasgow, Lothian, Borders and Fife were included in the study of women younger than 36 years, while only Thames, Oxford and Yorkshire, and only Oxford and Yorkshire Regions constituted catchments areas for the other two studies. The studies were restricted to white women with no previous malignancy (except nonmelanoma skin cancer), severe mental handicap or illness.

Cases were women with an incident invasive breast cancer, identified chiefly through the Regional Cancer Registries, and supplemented by hospital discharge records from Hospital Activity Analysis registers and patient lists kept at major treatment centres. Permission from hospital consultants and general practitioners was obtained before names were disclosed by the Cancer Registry and before women were contacted. Breast cancer was confirmed when case notes were reviewed in 97% of cases. Out of 1049, 838 and 635 eligible cases in the three studies of women less than 36, 36–45 and 46–54 years of age, 755 (72%), 644 (77%) and 535 (84%), respectively, were interviewed. Reasons for nonparticipation included consultants’ or general practitioners’ refusal (7, 6 and 2%), woman's own refusal (3, 3 and 2%), death (16, 11 and 9%) and relocation from the study area (3, 3 and 2%, respectively).

For each case, one age-matched control was randomly selected from the list of the case's general practitioner. If a chosen control could not be interviewed, a further age-matched control was selected at random. Of the 755, 644 and 535 first eligible controls in the studies of women less than 36, 36–45 and 46–54 years of age, 675 (89%), 588 (91%) and 491 (92%), respectively, were interviewed. Reasons for first controls’ nonparticipation included refusal by the general practitioners (2, 3 and 2% respectively) or the woman herself (8, 6 and 6% respectively).

### Data collection

Participants were interviewed in their homes using a standardised structured questionnaire, each case–control pair by the same trained interviewer, on average 2 years after diagnosis in each study. Women were asked to state whether they had been thin, average or plump at age 10 years, their weight at age 20 years and their weight and height at diagnosis (or equivalent). Participants were further asked about the number of hours spent per week in compulsory school sports at ages 12, 14, 16 and 18 years as well as the number of hours per week in all other sports at ages 12, 14, 16, 18, 20, 25, 30, 35, 40, 45 and 50 years, as appropriate. They were also asked about their menstrual history and other risk factors for breast cancer. The information requested, the structure of the questionnaire and phrasing of the questions was similar in the three studies.

### Classification of body fatness and participation in sports

Perceived body fatness at age 10 years was classified according to the women's responses as thin, average or plump. Body mass index (BMI) at age 20 years and at around the time of diagnosis was calculated as weight (kg) divided by the square of height (m); women were categorised as being thin, average or overweight if they had a BMI of <20.0, 20.0–22.5 or >22.5 kg m^−2^, respectively, at age 20 or <22.5, 22.5–25.0 or >25.0 kg m^−2^, respectively, at diagnosis. The subjects were also crossclassified by (a) their body fatness at age 10 years and their BMI at age 20 years and (b) their body fatness at age 10 years and their BMI at diagnosis, according to the following nine categories: thin/thin; thin/average; thin/overweight; average/thin; average/average; average/overweight; plump/thin; plump/average and plump/overweight.

We used the self-reported information on sports participation to create indices of the average number of hours per week of participation in compulsory school and other sports *at around puberty* (ages 12–14 years), *late adolescence* (ages 16–18 years), *early adulthood* (ages 20–30 years), *at around diagnosis* (the most recent 10-year interval) and during *all young ages* (ages 12–30 years).

### Statistical analyses

Women were defined as postmenopausal if they reported having had natural menopause (*n*=306), bilateral oophorectomy (*n*=32) or hysterectomy and were aged 48 years or older at diagnosis (*n*=154). All analyses were restricted to premenopausal women, with users of hormone replacement therapy in this group being further excluded (*n*=270). Thus, the final study group comprised 1560 cases and 1548 controls.

Conditional logistic regression was used for all analyses with stratification by age at diagnosis (single year) and region of recruitment. Odds ratios as estimates of relative risks (RRs) and 95% confidence intervals (CIs) are reported. Where more than two groups were compared, variances were estimated by treating the relative risks as floating absolute risks (FARs). This approach yields floated standard errors and floated confidence intervals (Easton *et al*, 1991; Plummer, 2004). Presentation of the results in this way enables valid comparisons between any two exposure groups, even if neither is the baseline.

All estimates were routinely adjusted for parity and age at first birth (nulliparous, parous and age at first birth <25, parous and age at first birth ⩾25), height (<162, ⩾162 cm), use of oral contraceptives (never user, user within the last 5 years, user more than 5 years ago), alcohol consumption (non-drinkers, drinkers), family history of breast cancer (no history, mother and/or sister with breast cancer) and socioeconomic status (nonmanual/professional, manual/unemployed/housewife). In addition to these standard factors, we further adjusted results for perceived body fatness at age 10 years, the number of hours of participation in sports at young ages and at diagnosis (0–1, 2–3, 4+ hours week^−1^), age at menarche (<13, 13+ years), time from menarche to onset of regular cycles (<1 year, 1+ years) and history of irregular menstrual cycles (no history, positive history). Correspondingly, we also adjusted results of early participation in sports for perceived body fatness at age 10 years (thin, average, plump), BMI at age 20 (<20, 20–22.5, 22.5+) years, BMI at diagnosis (<22.5, 22.5–25.0, 25.0+) years and menstrual characteristics as above.

Lastly, we explored whether the impact of body fatness at age 10 years and sports participation at young ages varied with levels of one another and with other factors including the standard set of adjustment variables and menstrual characteristics. Analyses were conducted using the data from all studies combined, since sensitivity analyses, estimating each model separately within each study, were not indicative of the associations being materially different between studies.

All analyses were performed using Stata version 8.1 (Stata Corporation, College Station, Texas, USA). All statistical significance levels (*P*-values) quoted are two sided.

## RESULTS

Characteristics of cases and controls are given in Table 1. Almost half were less than 36 years, about half were from nonmanual or professional social classes, most were parous, and most had used oral contraceptives.

**Figure 1:**
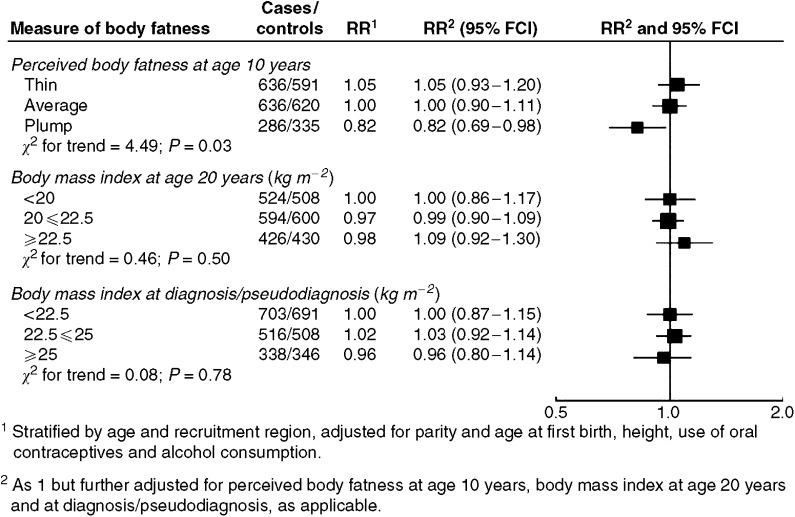
Relative risk of premenopausal breast cancer in relation to perceived body fatness at age 10 years, body mass index at age 20 years and at diagnosis/pseudodiagnosis. Black squares indicate relative risks (RRs), area of which is proportional to the amount of information contributed (i.e., to the inverse of the variance of the logarithm of the RRs). Lines indicate 95% floated confidence intervals (FCIs; see Material and Methods).

Among the controls, 40% perceived themselves as thin and 22% as plump at age 10 years. There was a statistically significant trend of a decreasing risk of breast cancer with increasing self-reported body fatness at age 10 years (*P*=0.03) (Figure 1). Women who reported having been plump at age 10 years had a borderline statistically significantly lower risk than women who reported having been of thin or average body build (RR=0.83, 95% CI 0.69–0.99). These associations were not affected by adjustment for BMI at age 20 years or for BMI at diagnosis (Figure 1).

Neither BMI at age 20 years nor BMI at diagnosis, within the narrow BMI range under study, was significantly related to risk among premenopausal women (Figure 1). Among controls, the mean BMI at age 20 years and at pseudodiagnosis were 21.5 and 23.3 kg m^−2^, respectively. Only 0.2 and 6.5% were obese (i.e. had a BMI ⩾30 kg m^−2^) at age 20 years and at diagnosis, respectively. The RR in the small subgroup of women who were obese at diagnosis was 0.78 (95% CI 0.56–1.06), as compared with those who had a BMI of less than 22.5 kg m^−2^. As compared with women who were under- or normal weight, those who reported to be overweight (i.e. had a BMI ⩾25 kg m^−2^) at age 20 years or at diagnosis were not at a significantly altered risk of breast cancer (RR=0.93 (95% CI 0.72–1.19) and 0.98 (95% CI 0.80–1.21), respectively).

Table 2 shows results for women crossclassified according to their perceived body fatness at age 10 years and their BMI at diagnosis. The lowest RR was among women who reported having been plump at age 10 years and who were overweight or obese at diagnosis (RR=0.75, 95% CI 0.54–1.01). No significant interactions were found between the effects of perceived body fatness at age 10 years and BMI at diagnosis (Table 2), nor between the effects of perceived body fatness at age 10 years and BMI at age 20 years (data not shown).

**Figure 2:**
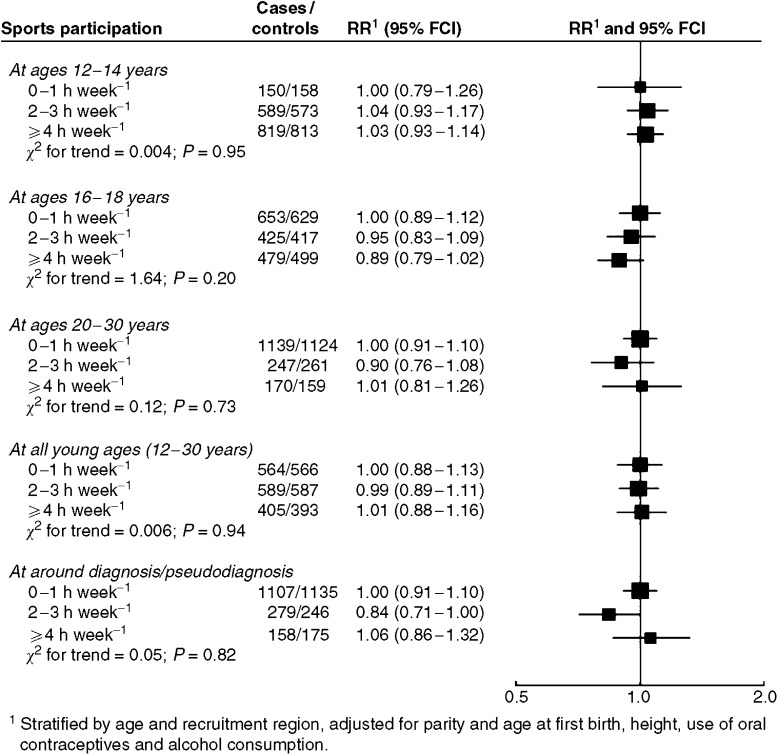
Relative risk of premenopausal breast cancer in relation to participation in sports at different ages. Black squares indicate relative risks (RRs), area of which is proportional to the amount of information contributed (i.e., to the inverse of the variance of the logarithm of the RRs). Lines indicate 95% floated confidence intervals (FCIs; see Material and Methods).

The average numbers of hours that controls reported engaging in sports, at ages 12–14, 16–18, 20–30 years, at all young ages (12–30 years) and at pseudodiagnosis were 4.7, 2.9, 1.3, 2.8 and 1.3 per week, respectively. Sports participation at these ages was not related to breast cancer risk (Figure 2). The RR for participating in sports for more than an hour each week from ages 12 to 30 years compared with less often was 1.00 (95% CI 0.86–1.16).

Further adjustment of perceived body fatness at age 10 for participation in sport or age at menarche and other menstrual factors, and participation in sport for body fatness at different ages or menstrual factors, did not alter the risk estimates (Table 3). Similarly, adjustments for age at menarche, other menstrual factors or participation in sports did not affect the associations between the risk of breast cancer, BMI at age 20 years and BMI at diagnosis (data not shown).

**Figure 3:**
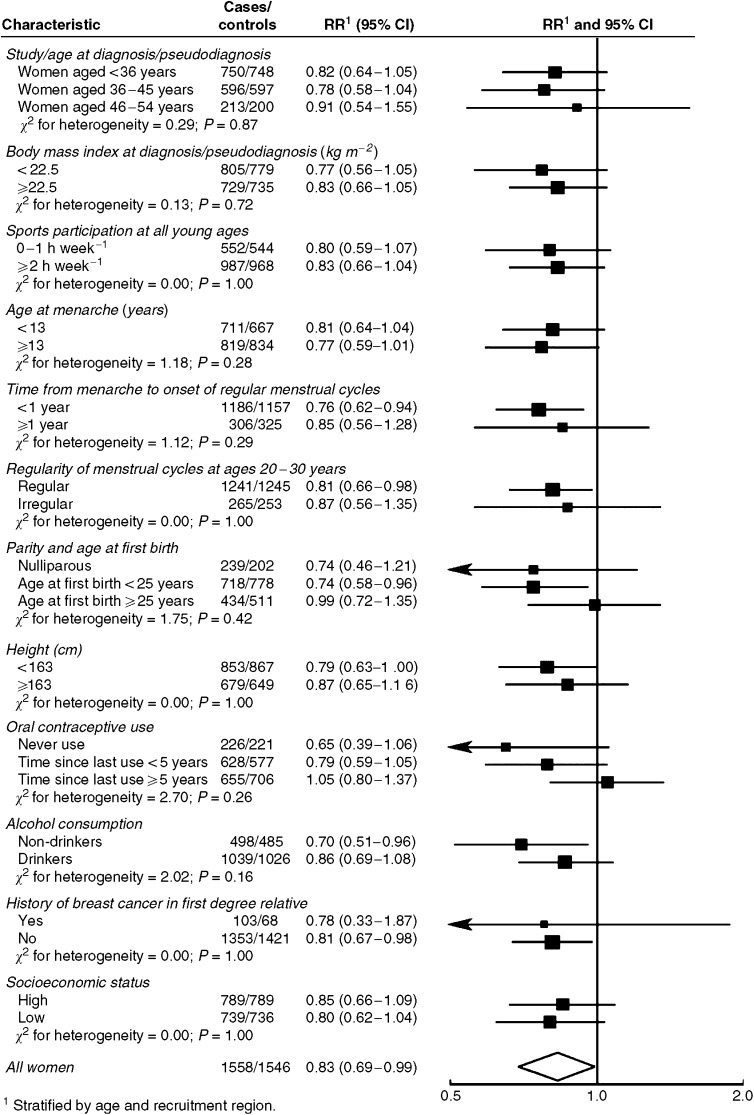
Relative risk for premenopausal breast cancer in women who perceived themselves as having been plump compared to thin or average at age 10 years, by different characteristics. Black squares indicate relative risks (RRs), area of which is proportional to the amount of information contributed (i.e., to the inverse of the variance of the logarithm of the RRs). Lines indicate 95% confidence intervals (CIs).

**Figure 4:**
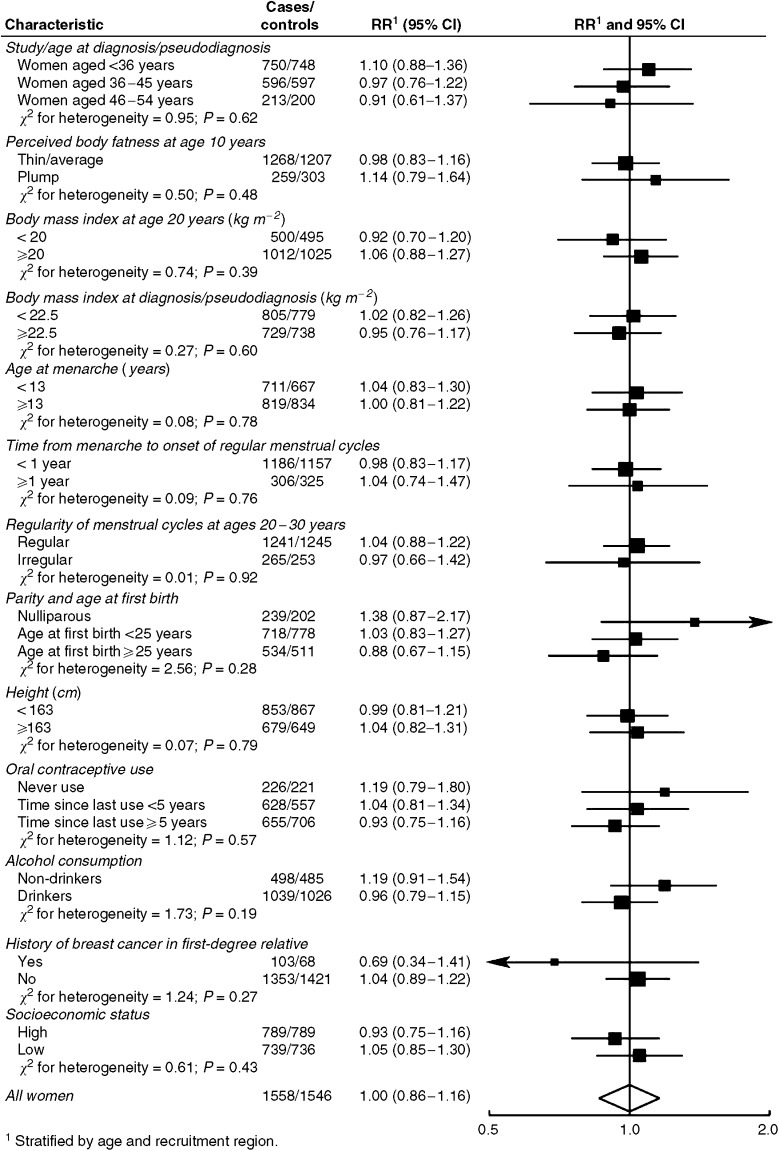
Relative risk for premenopausal breast cancer in women who reported participating in sports more than one compared to 1 h or less per week at ages 12–30 years, by different characteristics. Black squares indicate relative risks (RRs), area of which is proportional to the amount of information contributed (i.e., to the inverse of the variance of the logarithm of the RRs). Lines indicate 95% confidence intervals (CIs).

The observed relationships between breast cancer, perceived body fatness at age 10 years and participation in sports at ages 12–30 years did not vary significantly with levels of other characteristics (Figures 3 and 4). In some subgroups, however, the validity of the results is limited by the small number of observations.

## DISCUSSION

In this large, population-based case–control study of premenopausal women, three related measures of body fatness suggested that obesity in premenopausal women is associated with a reduced risk of breast cancer. Women who perceived themselves as plump at age 10 years had an RR of 0.83 (95% CI 0.69–0.99, *P*=0.03) compared with those who perceived themselves as thin. Self-reported obesity compared with leanness at diagnosis was associated with an RR of 0.78 (95% CI 0.56–1.06, *P*=0.11), and women who reported having been both plump at age 10 years and overweight at diagnosis had an RR of 0.75 (95% CI 0.56–1.01, *P*=0.06) as compared with those thin at age 10 years and at diagnosis. We did not find a relationship between any level of BMI at age 20 years and risk, but few women reported having been obese or even overweight at that age. The modest evidence of an inverse association between body fatness at age 10 years and risk of breast cancer among premenopausal women in our data is consistent with other reports (Berkey *et al*, 1999; Swerdlow *et al*, 2002; Okasha *et al*, 2003; Ahlgren *et al*, 2004; De Stavola *et al*, 2004; Weiderpass *et al*, 2004), as is the suggested protective effect of obesity at diagnosis (IARC, 2002). Although body fatness in childhood and early adulthood has been proposed to influence breast carcinogenesis via anovulatory-related progesterone deficiency, decelerated adolescent growth and oestrogen-induced breast epithelial differentiation (Berkey *et al*, 1999; Cabanes *et al*, 2004; Magnusson and Roddam, 2005), there is no direct evidence to support these mechanisms.

We did not observe any association between sports participation at puberty, late adolescence, early adulthood or at diagnosis and breast cancer risk in premenopausal women. The RR of breast cancer associated with an average of more than an hour each week of sports participation from ages 12 to 30 years, as compared with less often, was 1.00 (95% CI 0.86–1.16, *P*=0.98). Recent reviews concluded that physical activity both at young ages and in adulthood reduced the risk of breast cancer; IARC (2002) and Lagerros *et al* (2004). However, subsequent findings concerning premenopausal breast cancer are inconsistent, both for physical activity at young ages (Dorn *et al*, 2003; Steindorf *et al*, 2003; Yang *et al*, 2003; Margolis *et al*, 2005) and around diagnosis (Adams-Campbell *et al*, 2001; Gilliland *et al*, 2001; Matthews *et al*, 2001; Colditz *et al*, 2003; Dorn *et al*, 2003; Hirose *et al*, 2003; John *et al*, 2003; Steindorf *et al*, 2003; Yang *et al*, 2003; Margolis *et al*, 2005). Without evidence from a formal meta-analysis using individual data from all relevant studies, it is difficult to interpret the available results.

This is the largest study of premenopausal breast cancer on body fatness and sports participation at young ages with an over 80% power to detect an RR of 0.77 or less for at least 4 h week^−1^ of sports participation at young ages. It is population based, and had high participation rates among both cases and controls. It included detailed information about body fatness at different ages, and life-course physical activity including its duration, as well as about potential confounding factors. Nevertheless, differential and nondifferential misclassification may limit the validity of findings based on retrospective assessment of exposures. In addition, we lacked information on types of physical activity other than sports (for example, occupational physical activity, housework, gardening and transportation). However, sports participation could, however, be assumed to account for the major proportion of physical activity at young ages.

In summary, this large study provides evidence of an inverse association between body fatness, but not physical activity, at young ages and the risk of breast cancer in premenopausal women.

## Figures and Tables

**Table 1:** Descriptive characteristics of cases and controls

	**Cases (%)**	**Controls (%)**
**Characteristic**	***n*=1560**	***n*=1548**
*Age at diagnosis/pseudodiagnosis (years)*		
Study 1, women aged <36 years	751 (48)	748 (48)
Study 2, women aged 36–45 years	596 (38)	598 (39)
Study 3, women aged 46–55 years	213 (14)	202 (13)
		
*Socioeconomic status*		
Nonmanual/professional	809 (52)	804 (52)
Manual/unemployed/housewife	751 (48)	744 (48)
*Use of oral contraceptives*		
Never	250 (16)	259 (17)
Last use <5 years ago	663 (41)	567 (37)
Last use ⩾5 years ago	647 (43)	722 (47)
*Alchohol consumption*		
Non-drinkers	516 (33)	516 (34)
Drinkers	1047 (67)	1032 (66)
*History of breast cancer in first-degree relative*		
No	1357 (89)	1425 (94)
Yes	165 (11)	86 (6)
Mean height (cm)	163.3	162.3
Mean age at menarche (years)	12.6	12.7
Mean time from menarche to onset of regular menstrual cycles (months)	11	11
*Parity*		
Nulliparous	270 (17)	230 (15)
Parous	1290 (83)	1318 (85)
Mean age at first birth, years (among parous)	24	23.8
*History of irregular menstrual cycles*		
Yes	306 (20)	287 (19)
No	1248 (80)	1256 (81)

**Table 2:** Relative risk of premenopausal breast cancer in relation to change in body fatness between age 10 years and diagnosis/pseudodiagnosis

**Body fatness at age 10 years^a^ and at diagnosis/pseudodiagnosis^b^**	**Cases/controls**	**RR^c^**	**95% FCI^d^**
Thin/thin	377/341	1.00	0.86–1.16
Thin/average	162/166	0.89	0.72–1.12
Thin/overweight	97/84	1.04	0.77–1.40
Average/thin	254/272	0.84	0.71–1.00
Average/average	249/219	1.05	0.88–1.27
Average/overweight	133/130	0.92	0.72–1.18
Plump/thin	73/79	0.85	0.61–1.17
Plump/average	105/124	0.79	0.61–1.03
Plump/overweight	108/132	0.75	0.58–0.97

a Perceived body fatness at age 10 years.

b Body fatness at diagnosis in cases and at an equivalent time in controls, based on body mass index (BMI) (thin=BMI<20 kg m^−2^, average=BMI 20–25 kg m^−2^ and overweight=BMI⩾25 kg m^−2^).

c Relative risk, compared with thin/thin women, stratified by age and recruitment region, adjusted for parity, age at first birth, height, use of oral contraceptives and alcohol consumption.

d Floated confidence intervals (see Material and Methods).

**Table 3:** Relative risk of premenopausal breast cancer in relation to (A) perceived body fatness at age 10 years and (B) participation in sports at young ages, with and without adjustment for body fatness, participation in sports at different ages and menstrual characteristics

	**Thin**		**Average**		**Plump**	
**(A) Model/perceived body fatness at age 10 years**	**RR^a^**	**95% FCI^b^**	**RR^a^**	**95% FCI^b^**	**RR^a^**	**95% FCI^b^**
Multivariate-adjusted^c^	1.05	0.94–1.17	1.00	0.89–1.12	0.85	0.72–0.99
Multivariate^c^+participation in sports at all young ages	1.05	0.93–1.17	1.00	0.89–1.12	0.84	0.72–0.99
Multivariate^c^+participation in sports at diagnosis/pseudodiagnosis	1.04	0.93–1.17	1.00	0.89–1.12	0.84	0.72–0.99
Multivariate^c^+menstrual characteristics^d^	1.06	0.95–1.20	1.00	0.89–1.12	0.84	0.72–0.99
						

	**0–1 h week^−1^**		**2–3 h week^−1^**		**4+ h week^−1^**	
**(B) Model/average duration of sports participation at ages 12–30 years**	**RR^e^**	**95% FCI^b^**	**RR^e^**	**95% FCI^b^**	**RR^e^**	**95% FCI^b^**
Multivariate-adjusted^c^	1.00	0.90–1.13	0.99	0.89–1.11	1.01	0.88–1.16
Multivariate^c^+body fatness at age 10	1.00	0.88–1.13	0.99	0.88–1.11	1.00	0.87–1.16
Multivariate^c^+body mass index (BMI) at age 20 years	1.00	0.88–1.13	0.98	0.87–1.10	1.00	0.86–1.14
Multivariate^c^+BMI at diagnosis/pseudodiagnosis	1.00	0.88–1.13	0.99	0.88–1.11	1.01	0.87–1.16
Multivariate^c^+menstrual characteristics^d^	1.00	0.88–1.13	0.98	0.88–1.10	1.00	0.87–1.15

a Relative risk, compared with women who reported to be of average body fatness at age 10 years.

b Floated confidence intervals (see Material and Methods).

c Stratified by age and recruitment region, adjusted for parity, age at first birth, height, use of oral contraceptives and alcohol consumption.

d Additional adjustments for age at menarche, time from menarche to onset of regular cycles and irregular menstrual cycles at ages 20–30 years.

e Relative risk, compared with women who reported to participate 1 h or less per week in sports at young ages.
